# *Neisseria gonorrhoeae* Infections

**DOI:** 10.3390/pathogens9080647

**Published:** 2020-08-12

**Authors:** Beatriz Suay-García, María-Teresa Pérez-Gracia

**Affiliations:** 1 ESI International Chair@CEU-UCH, Departamento de Matemáticas, Física y Ciencias Tecnológicas, Universidad Cardenal Herrera-CEU, CEU Universities, San Bartolomé 55, 46115 Alfara del Patriarca (Valencia), Spain; beatriz.suay@uchceu.es; 2 Área de Microbiología. Departamento de Farmacia, Universidad Cardenal Herrera-CEU, CEU Universities, C/Ramón y Cajal s/n, 46115 Alfara del Patriarca (Valencia), Spain

**Keywords:** *Neisseria gonorrhoeae*, sexually transmitted infection, transmission, diagnosis, antibiotic treatment

## Abstract

Gonorrhea is a sexually transmitted disease with a high morbidity burden. Despite having guidelines for its treatment, the incidence of the disease follows an increasing trend worldwide. This is mainly due to the appearance of antibiotic-resistant strains, inefficient diagnostic methods and poor sexual education. Without an effective vaccine available, the key priorities for the control of the disease include sexual education, contact notification, epidemiological surveillance, diagnosis and effective antibiotic treatment. This Special Issue focuses on some of these important issues such as the molecular mechanisms of the disease, diagnostic tests and different treatment strategies to combat gonorrhea.

*Neisseria gonorhoeae* is an obligate human pathogen that causes gonorrhea, a sexually transmitted disease (STD). This Gram-negative diplococcus is highly infective due to its virulence factors: pili, Por proteins, Opa proteins, Rmp proteins, lipooligosaccharides and IgA protease. The most common form of presentation in men is acute anterior urethritis, while gonococcal infection in women does not have specific symptoms. Although the prevailing view is that infections in women are mainly asymptomatic whereas infections in men are not, many studies show that asymptomatic infections are prevalent in both sexes. Gonorrhea is primarily transmitted from an infected individual by direct human-to-human contact between the mucosal membranes of the urogenital tract, anal canal and the oropharynx, usually during sexual activities.

Gonorrhea is a community disease with a high morbidity burden, representing 88 million of the estimated 448 million new cases of curable STDs that occur yearly worldwide [1]. Furthermore, the incidence of the disease is rising due to the prevalence of multidrug-resistant strains [2]. In fact, the appearance of resistances in this microorganism threatens the effectiveness of the available gonorrhea treatments to such extent that it has been classified as a “Priority 2” microorganism in the WHO *Global Priority List of Antibiotic-Resistant Bacteria to Guide Research, Discovery, and Development of New Antibiotics* [3]. Ever since sulphonamides were introduced to treat gonorrhea in the 1930s, gonococci have continuously shown an extraordinary ability to develop resistance to any antimicrobial introduced for treatment [4]. Treatment is currently given empirically, without performing antimicrobial susceptibility tests. However, the increasing issue of drug-resistant gonococci has led the scientific community to focus research on new drugs and alternative treatments, which has obtained encouraging results. The diagnosis of gonorrhea is established by identification of *N. gonorrhoeae* in genital, rectal, pharyngeal or ocular secretions. *N. gonorrhoeae* can be detected by culture or nucleic acid amplification tests and, in some cases, Gram staining. Without an effective vaccine available, the key priorities for the prevention and control of the disease include public health and sexual education, contact notification, epidemiological surveillance, early diagnosis and effective antibiotic treatment.

In this Special Issue, which is devoted to understanding some of the important issues about *Neisseria gonorrhoeae* infection, there are six contributions in the form of original research papers, review articles and a brief report focused on the physiopathology of gonorrhea, diagnostic tests and different treatment strategies against this STD. More specifically, the first research article focuses on the identification of an *N. gonorrhoeae* histone deacetylase [5]. The research conducted shows that the presence of this enzyme during gonococcal infection reduces the expression of host defense peptides and stimulates promoters of pro-inflammatory mediator genes. These discoveries suggest that gonococci can exert epigenetic modifications on host cells to modulate macrophage defense genes, leading to a poorer trained immunity response.

The second research article assesses ciprofloxacin susceptibility in strains obtained from patients attending STD clinics to receive treatment [6]. The strains isolated in STD clinics in Baltimore (USA) had an overall ciprofloxacin resistance prevalence of 32.4% when evaluated by Gyrase A PCR and E-test. It must be noted that this percentage increased over the years studied, from an initial 24.7% in 2014 to 45.2% in 2016. Researchers conclude that, in this environment, ciprofloxacin could be used as a targeted treatment. However, they highlight that point-of-care tests for *N. gonorrhoeae* diagnosis and susceptibility testing are urgently needed to identify individuals who can be treated with this targeted approach.

Furthermore, three insightful review articles discuss relevant topics such as the assessment of the risks of relying on antibiotics to reduce gonococcal prevalence by analyzing historical data on the appearance of antibiotic resistance after mass-meningococcal campaigns [7]; the different laboratory diagnostic options available and future options for a more efficient and affordable diagnosis [8]; and the diversity of the genus *Neisseria* in the clinical context, bringing attention to the many pathologies these species may cause [9]. Lastly, a brief report analyzes the transmission of commensal *Neisseria* between sexual partners and the implications this may have in the transmission of antibacterial resistances [10].
